# Absence of Appl2 sensitizes endotoxin shock through activation of PI3K/Akt pathway

**DOI:** 10.1186/2045-3701-4-60

**Published:** 2014-10-02

**Authors:** Liufeng Mao, Wanhua Lin, Tao Nie, Xiaoyan Hui, Xuefei Gao, Kuai Li, Mengxiao Ding, Xiaofeng Tang, Peng Li, Yu Wang, Aimin Xu, Pentao Liu, Donghai Wu

**Affiliations:** Key Laboratory of Regenerative Biology, Guangzhou Institute of Biomedicine and Health, Chinese Academy of Sciences, Guangzhou, 510530 China; School of Life Sciences, Guangxi Normal University, Guilin, 541004 China; Lab for Stem Cell and Pharmaceutical Biotechnology of Guangxi Normal University, Guilin, 541004 China; State key laboratory of pharmaceutical biotechnology, Department of Medicine, The University of Hong Kong, Hong Kong, 999077 China; Wellcome Trust Sanger Institute, Cambridge, CB10 1SA UK

**Keywords:** Appl2, Appl1, Endotoxin shock, Inflammatory cytokines, PI3K, Akt, NF-κB

## Abstract

**Background:**

The adapter proteins Appl1 (adaptor protein containing pleckstrin homology domain, phosphotyrosine domain, and leucine zipper motif 1) and Appl2 are highly homologous and involved in several signaling pathways. While previous studies have shown that Appl1 plays a pivotal role in adiponectin signaling and insulin secretion, the physiological functions of Appl2 are largely unknown.

**Results:**

In the present study, the role of Appl2 in sepsis shock was investigated by using Appl2 knockout (KO) mice. When challenged with lipopolysaccharides (LPS), Appl2 KO mice exhibited more severe symptoms of endotoxin shock, accompanied by increased production of proinflammatory cytokines. In comparison with the wild-type control, deletion of Appl2 led to higher levels of TNF-α and IL-1β in primary macrophages. In addition, phosphorylation of Akt and its downstream effector NF-κB was significantly enhanced. By co-immunoprecipitation, we found that Appl2 and Appl1 interacted with each other and formed a complex with PI3K regulatory subunit p85α, which is an upstream regulator of Akt. Consistent with these results, deletion of Appl1 in macrophages exhibited characteristics of reduced Akt activation and decreased the production of TNFα and IL-1β when challenged by LPS.

**Conclusions:**

Results of the present study demonstrated that Appl2 is a critical negative regulator of innate immune response via inhibition of PI3K/Akt/NF-κB signaling pathway by forming a complex with Appl1 and PI3K.

## Background

Severe sepsis, or septic shock, represents one of the oldest problems in medicine. In the United States, severe sepsis composes 2% of patients admitted to the hospital [1]. Septic shock is caused by severe infection of the invading microbes that produces endotoxin from Gram-negative bacteria or analogous molecules from gram-positive bacteria or other fungi. Endotoxins are bacterial membrane lipopolysaccharides (LPS) and are the major component of the outer membranes in Gram-negative bacteria. In Gram-negative sepsis, LPS (endotoxin) induces profound activation of macrophages and production of potent inflammatory cytokines such as TNF-α, IL-1 and IL-6 [2]. These proinflammatory cytokines act on endothelial cells to cause systemic vasodilatation, diminished myocardial contractility, endothelial injury and activation, resulting in disseminated intravascular coagulation. The hypo-perfusion in turn leads to multi-organ failure that affects the liver, kidneys, and central nervous system.

LPS elicits its biological effects by forming a complex with CD14, LBP, TLR4, and MD-2, which triggers several intracellular signal pathways to activate NF-κB [3]. NF-κB is a transcriptional factor containing five subunits, namely, relA/p65, relB, c-Rel, p105/p50, and p100/p52, which function as homo- or hetero-dimers. In resting macrophages, NF-κB dimers reside in the cytoplasm and bind with specific inhibitory IκB proteins. When stimulated by LPS, IκB kinase (IKK) phosphorylates IκB, leading to its degradation, thereby releasing NF-κB for nuclear translocation where it facilitates the expression of various inflammatory and stress response genes [4–7]. Jun N-terminal kinase, p38 mitogen-activated protein kinase, and Akt pathways have been reported to activate NF-κB. Among these pathways, Akt stimulates NF-κB by phosphorylating and activating the NF-κB p65/RelA subunit through the PI3K/Akt/IKK/IκB/NF-κB signaling cascade [8–12].

Appl1 and Appl2 are homologous proteins that bind to a diverse set of transmembrane receptors or signaling proteins. Appl1 was originally identified as an intracellular binding partner of adiponectin receptor and mediates adiponectin-dependent insulin sensitization in skeletal muscle [13, 14]. Appl1 plays an essential role in inflammatory responses, depending on the cell type. Specifically, adiponectin was shown to either suppress NF-kB activity in endothelial cells and adipocytes or to activate the NF-kB pathway in synovial or cardiac fibroblasts [15–20]. Appl1 also participates in PI3K/Akt signaling pathway since it was reported to induce the phosphorylation of Akt and to stimulate the transactivation of the p65 subunit of NF-κB through the IKK [21]. Appl1 transgenic mice showed less peripheral insulin resistance and cardiac dysfunction in response to high fat diet with enhanced Akt phosphorylation and glucose uptake in cardiomyocyte [22]. Additionally, Tan et al. has reported that Appl1 possesses growth factor-selective effects on Akt signaling in mouse embryonic fibroblasts [23].

On the other hand, the physiological functions of Appl2 are less clear. Whether Appl2 plays a role in inflammatory responses has not yet been studied. However, since it was demonstrated by several subsequent studies that Appl2 down-regulated adiponectin signaling by forming a Yin-Yang regulatory pair with Appl1, it is important to examine whether Appl2 plays a negative regulatory role in inflammation [24, 25].

In this study, Appl2 knockout (KO) mouse was generated and its response to LPS-induced endotoxin shock was investigated. When challenged with LPS, Appl2 KO mice had more exacerbated symptoms of endotoxin shock. In accordance with this, circulating levels and production of proinflammatory cytokines in Appl2 KO mice and macrophages were markedly elevated in comparison to their controls. Further analysis demonstrated that Appl2 modulated Akt-NF-κB signaling pathway, possibly by competitively binding to the p85 subunit of PI3K. Results of the present study suggest that Appl2 functions as a negative regulator of innate immune response via Akt pathway.

## Results

### Generation of Appl2 KO mice

**Figure 1:**
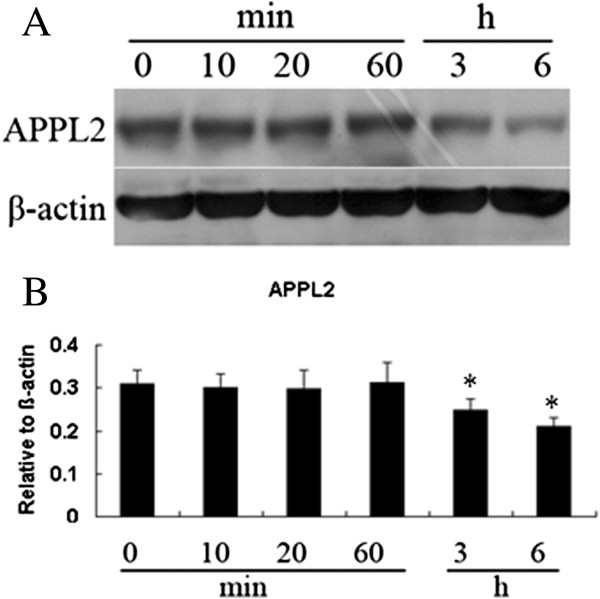
**Decreased expressions of Appl2 in primary macrophages treated with LPS. (A)** Western blot analysis of Appl2 in peritoneal macrophages from WT mice stimulated with 1 μg/mL LPS at specified time points. **(B)** The density analysis of the above data using NIH Image J software for relative values. *p < 0.05, compared with control, n = 5.

**Figure 2:**
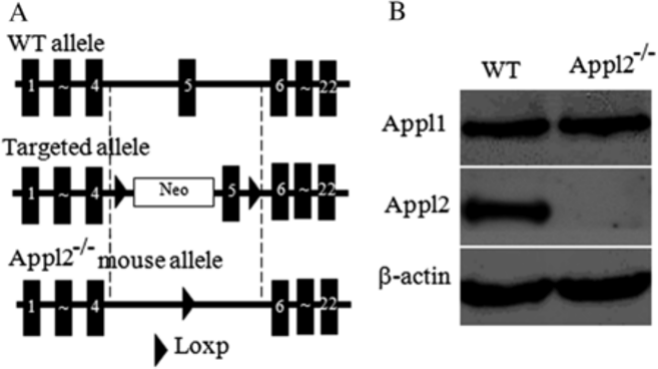
**Generation and validation ofAppl2 KO mice. (A)** Diagram showing the construction of Appl2 KO mouse. **(B)** Western blot analysis demonstrating the absence of the Appl2 protein in the macrophages of Appl2 KO mice. Appl2^−/−^, Appl2 KO mouse.

To study the role of Appl2 in inflammatory responses, the expression of Appl2 in mouse primary macrophages was examined during acute LPS stimulation. The expression of Appl2 decreased steadily (Figure 1) in response to LPS, suggesting that Appl2 may have distinct functions in the inflammatory responses. To better understand its role in inflammation, Appl2 KO mice were generated as described in the method. The deletion of Appl2 in these mice was confirmed by Western blot analysis in primary peritoneal macrophages (Figure 2).

### Appl2 KO mice are more prone to endotoxic shock when challenged by LPS

**Figure 3:**
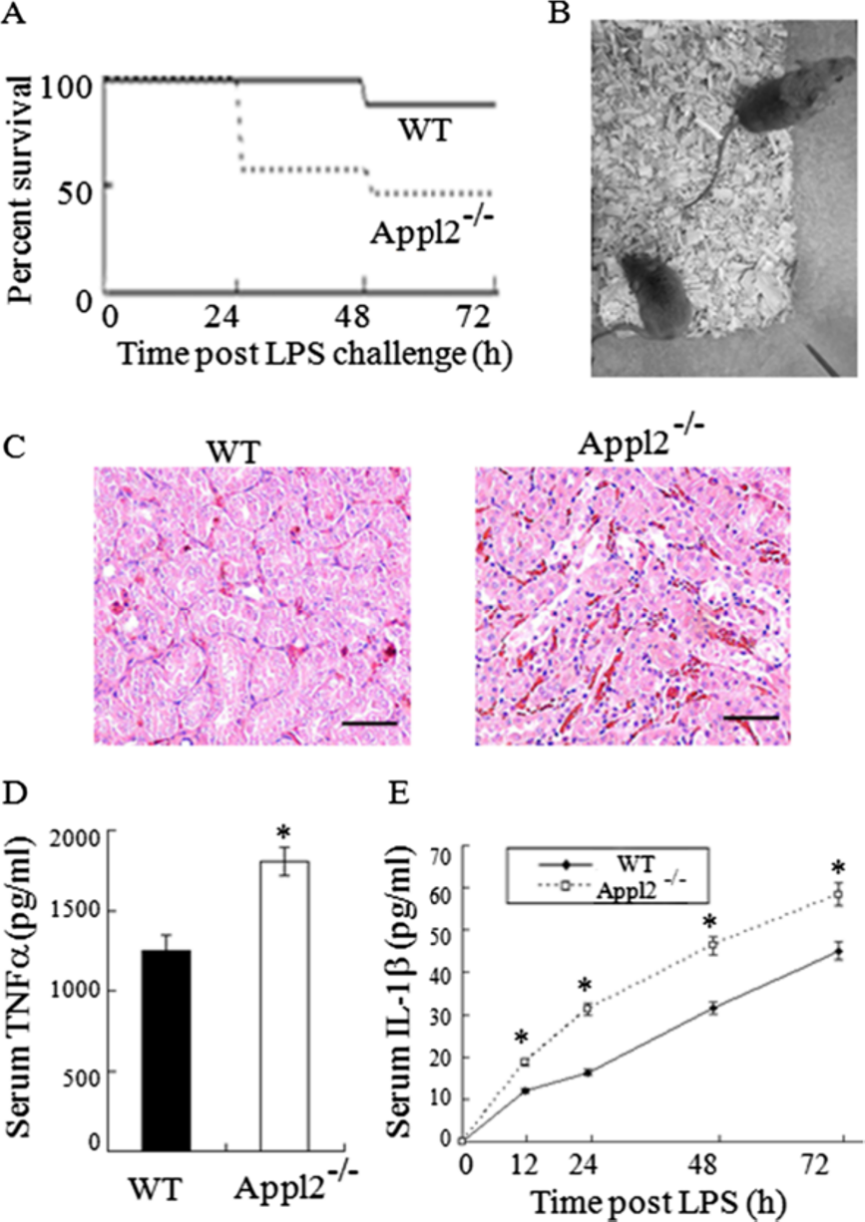
**Appl2 KO mice are more prone to endotoxic shock than WT mice. (A)** Kaplan-Meier survival curves of the WT (n = 9) and the Appl2 KO (n = 9) mice after LPS challenge (20 mg/kg weight, intraperitoneal injection). Compared with the WT mice, the Appl2 KO mice were more susceptible to the endotoxin challenge. **(B)** Photographs of the WT and Appl2 KO mice 24 h after intraperitoneal injection of LPS. WT mice showed only mild effects, whereas the Appl2 KO mice exhibited typical signs of endotoxemia. **(C)** HE-stained kidney sections from mice at 24 h post-LPS challenge. **(D)** Mouse serum TNFα level at 60 min after LPS injection. **(E)** Time course of the IL-1β expression in the LPS-stimulated Appl2 KO and control mice. Scale bar: 50 μm; *p < 0.05, compared with WT mice, n = 6.

To examine the roles of Appl2 in regulating inflammatory responses, the Appl2 KO mice were subjected to LPS challenge and the phenotypes were examined. The mice were intraperitoneally injected with indicated doses of LPS, and the survival rate of the mice was monitored. At a dose of 20 mg /kg LPS, the Appl2 KO mice showed more severe symptoms of endotoxin shock and higher mortality rate compared with the WT mice (Figure 3A-B). Survival analysis showed that the survival rate of the Appl2 KO mice was significantly lower than that of the WT mice after injection of LPS.A critical feature of endotoxic shock is disseminated intravascular coagulation, which is characterized by widespread blood coagulation and vessel hemorrhage, particularly in the kidney. After challenge with the indicated doses of LPS, a histological examination of the kidneys was performed. Appl2 KO mice exhibited server hemorrhage in their kidneys when the WT mice did not show any obvious tissue damage at the dose of LPS (Figure 3C). The TNFα and IL-1β serum levels were subsequently measured since TNFα and IL-1β are critical proinflammatory cytokines that respond to endotoxic shock elicited by LPS. Compared with the WT mice, the serum levels of TNFα and IL-1β were higher in the Appl2 KO mice (Figure 3D-E).

### Increased production of TNFα and IL-1β in Appl2 KO macrophages

**Figure 4:**
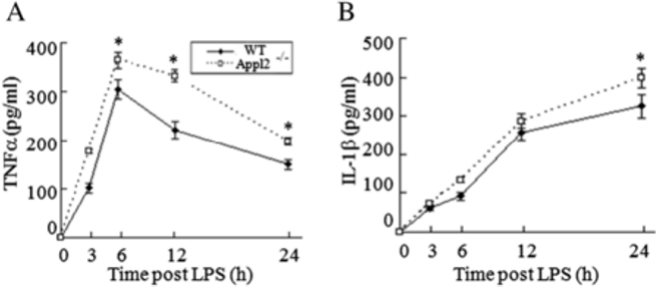
**Increased production of TNFα and IL-1β in Appl2 KO macrophages. (A)** Kinetics of TNFα expression in the LPS-stimulated primary macrophages of the Appl2 KO and control mice. **(B)** Kinetics of IL-1β expression in the LPS-stimulated primary macrophages of the Appl2 KO and control mice. *p < 0.05, compared with WT mice, n = 5.

Macrophages are the major target cells of LPS stimulation that produce proinflammatory cytokines; thus, the peritoneal primary macrophages were harvested and stimulated with LPS *in vitro*. After LPS stimulation, the production of cytokines secreted into the medium in the primary macrophages was determined by ELISA. The kinetics of TNF-α and IL-1β cytokine expressions were monitored. Compared with the macrophages isolated from WT mice, the production of TNF-α and IL-1β was significantly enhanced in macrophages from Appl2 KO mice upon LPS stimulation (Figure 4A-B). These results suggest that loss of Appl2 enhances the proinflammatory cytokine in the primary macrophage in response to LPS challenge.

### Enhanced activation of Akt-NF-κB pathways after LPS stimulation in Appl2 KO macrophages

**Figure 5:**
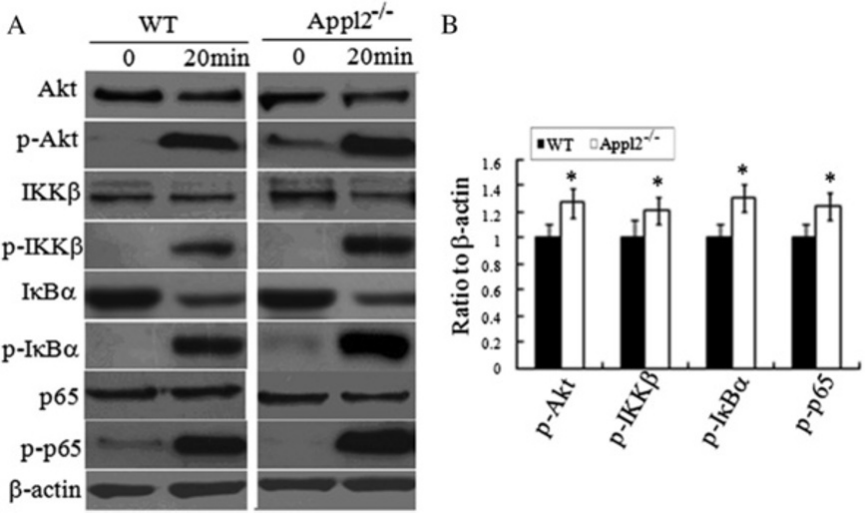
**Enhanced activation of Akt-NF-κB pathways after LPS stimulation in Appl2 KO macrophages.** Peritoneal macrophages derived from peritoneal cells were stimulated with 1 μg/mL LPS for 20 min. The cell lysates were analyzed by western blot. **(A)** Western blot analysis on peritoneal macrophages from APPL2 KO mice with the indicated antibodies. **(B)** The ratio values of p-Akt/Akt/β-actin, p-IKKβ/IKKβ/β-actin, p-IκB/ IκB/β-actin, p-p65/p65/β-actin as determined by the density analysis of **(A)** using NIH Image J software for relative values. *p < 0.05, compared with WT mice, n = 5.

Thus, whether LPS can activate the Akt pathway was first examined. In Figure 5, compared with the LPS-stimulated WT mice macrophage, phospho-Akt was enhanced in Appl2 KO mice macrophages. Subsequently, the activation of the p65 subunit of NF-κB was also examined, which can be regulated through the Akt pathway. In macrophages from Appl2 KO mice, phospho-IKKβ, IκB, and p65 were enhanced compared with the WT macrophages (Figure 5). These data suggest that the Appl2 can regulate inflammation through the Akt pathway for NF-κB activation.

### The p85 subunit of PI3K associates with Appl2 and Appl1

**Figure 6:**
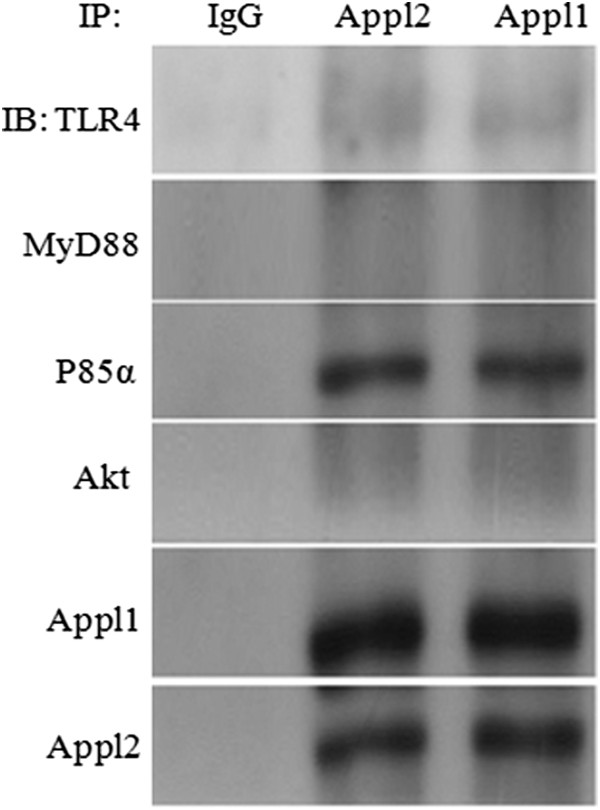
**The p85α subunit of PI3K associates with Appl 1 and 2.** Peritoneal macrophages from WT mice were treated with 1 μg/mL LPS for 20 min. Macrophages lysates were immunoprecipitated with IgG, Appl2 and Appl1 antibody and immuoblotted using antibodies against TLR4, MyD88, Akt, p85α, Appl1 and Appl2, respectively.

Appl isoforms interact with many different membrane receptors and others proteins. MyD88 dependent toll-like receptor 4 (TLR4)signaling pathway is a major receptor for LPS in mediating innate immune response [26]. To further pin down the molecular mechanism by which Appl2 modulates inflammation, we first examined whether there exists an interaction between Appl2with TLR4 and MyD88. Immunoprecipitation results demonstrated that Appl2 has weak interaction with TLR4 but no interaction with MyD88. Moreover, Appl2 was found to strongly interact with p85α subunit of PI3K and Appl1. Meanwhile we also found Appl1 could interact with p85α subunit of PI3K and Appl2 (Figure 6).

### Appl1 KO mice show less severe endotoxic shock than WT mice

**Figure 7:**
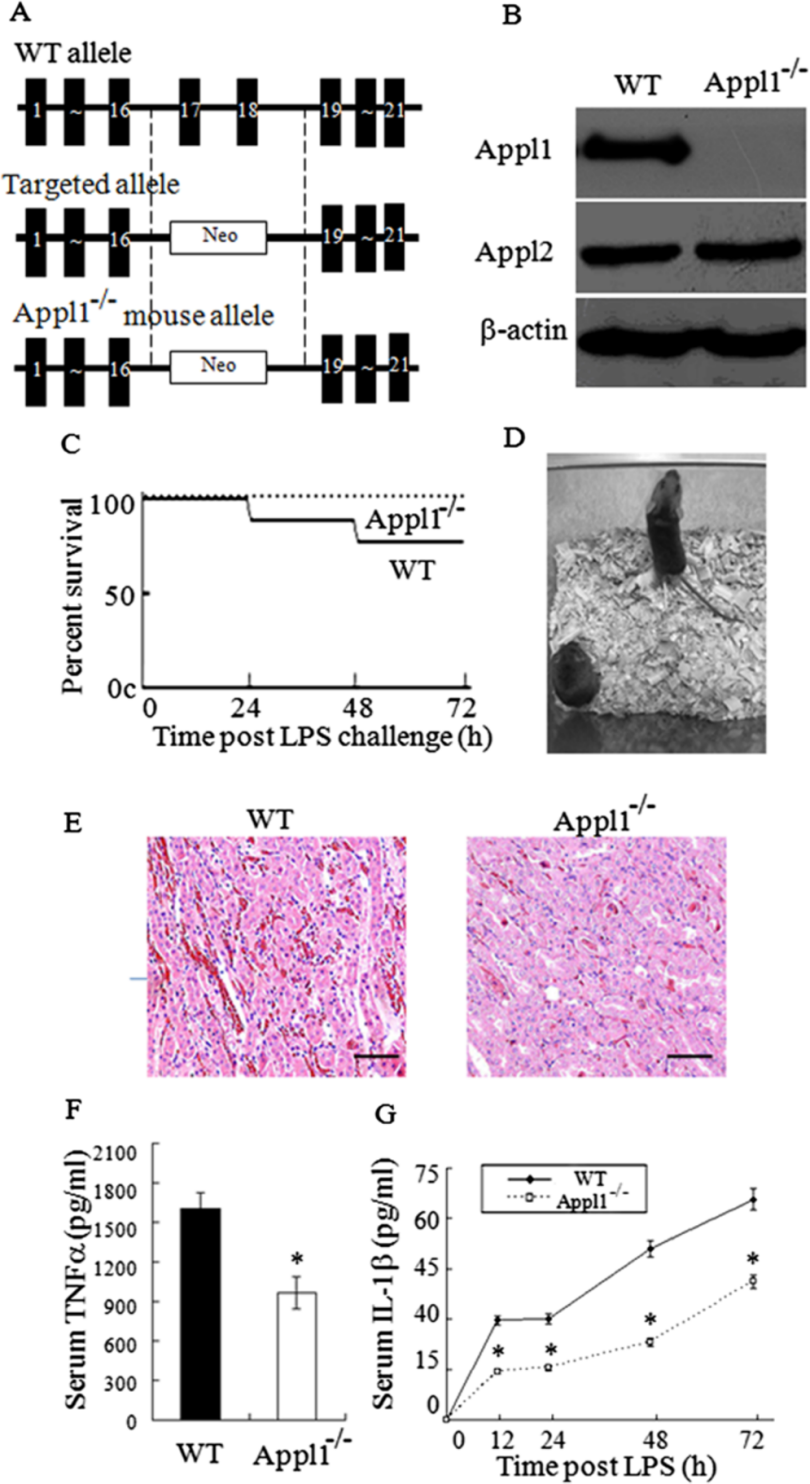
**Appl1 KO mice show less severe endotoxic shock than WT mice. (A)** Diagram showing the construction of Appl1 KO mouse. **(B)** Western blot analysis demonstrating the absence of the Appl1 protein in the macrophages of Appl1KO mice. **(C)** Kaplan-Meier survival curves of the WT (n = 9) and the Appl1 KO (n = 9) mice after LPS challenge (30 mg/kg weight, intraperitoneal injection). Compared with the WT mice, the Appl1 KO mice were less susceptible to the endotoxin challenge. **(D)** Photographs of the WT and Appl1 KO mice 24 h after intraperitoneal injection of LPS. Appl1 KO mice showed only mild effects, whereas WT mice exhibited typical signs of endotoxemia. **(E)** HE-stained kidney sections from mice at 24 h post-LPS challenge. **(F)** Mouse serum TNFα level at 60 min after LPS injection. **(G)** Kinetics of the IL-1β expression in the LPS-stimulated Appl1 KO and control mice. Scale bar: 50 μm; Appl1^−/−^, Appl1 KO mouse; *p < 0.05, compared with WT mice, n = 6.

To better understand their roles in inflammation, Appl1 KO mice were generated (Figure 7A-B). At 30 mg/kg LPS challenge, the Appl1 KO mice showed less severe endotoxic shock and lower mortality compared with the WT mice (Figure 3C-D). After the indicated doses of LPS challenge, a histological examination of the kidneys was performed. Compared with the WT mice, the Appl1 KO mice showed less widespread hemorrhage in their kidneys (Figure 7E), and lower production of TNFα and IL-1β in serums (Figure 7F-G).

### Reduced production of TNFα and IL-1β and decreased activation of Akt-NF-κB pathways after LPS stimulation in Appl1 KO macrophages

**Figure 8:**
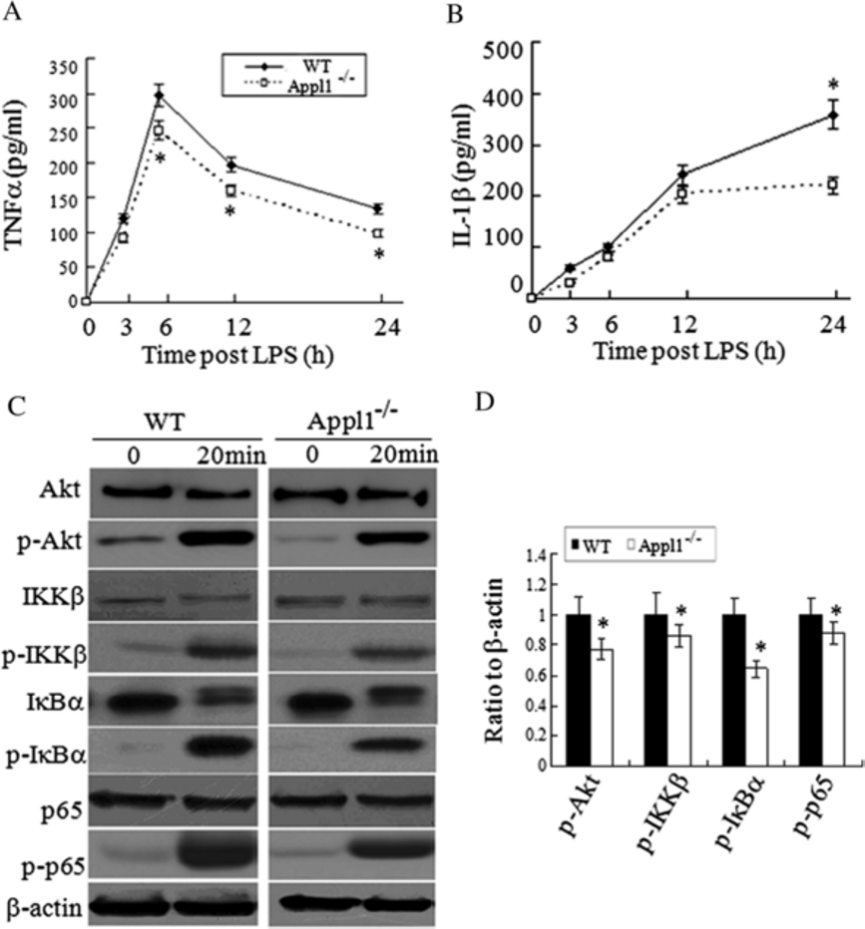
**Decreased production of TNFα and IL-1β and reduced activation of Akt-NF-κB pathways after LPS stimulation in Appl1 KO macrophages.** Peritoneal macrophages derived from peritoneal cells were stimulated with 1 μg/mL LPS at specified times and the supernant was collected for quantification of cytokine (TNFα and IL-1β) expression. The cell lysates were analyzed by western blot. **(A-B)** Time course of TNFα and IL-1β expression in the LPS-stimulated primary macrophages from the Appl1 KO and control mice. **(C)** Western blot analysis on peritoneal macrophages from Appl1 KO mice with the indicated antibodies. **(D)** The ratio values of p-Akt/Akt/β-actin, p-IKKβ/IKKβ/β-actin, p-IκB/IκB/β-actin, and p-p65/p65/β-actin as determined by the density analysis of **(C)** using NIH Image J software for relative values. *p < 0.05, compared with WT mice, n = 5.

The kinetics of TNFα and IL-1β cytokine expressions was also examined to monitor the dynamic change in cytokine levels at designated periods after challenged by LPS. Compared with the macrophages from WT mice, the production of TNFα and IL-1β reduced significantly in the LPS-stimulated macrophages from the Appl1 KO mice (Figure 8A-B). Appl1 was mainly identified to interact with Akt and increase the phosphorilation of Akt. Phosphor-Akt can stimulate the transactivation of the p65 subunit of NF-κB through the IκB kinase. Thus, whether LPS can activate the Akt pathway was examined. In Figure 8C-D, compared with the LPS-stimulated WT mice macrophage, phosphor-Akt was attenuated in Appl1 KO mice macrophages. Phosphor-IKKβ, IκB, and p65 were also reduced compared with the WT mice macrophages. These data suggest that the Appl1 can regulate inflammation through the Akt pathway for NF-κB activation.

## Discussion

Mounting evidence demonstrates that App11 is an important mediator of insulin sensitivity and inflammatory response in adiponectin signaling pathways [27]. However, the cellular functions of Appl2 and its relationships with App11 remain poorly understood [24]. In order to examine the possible involvement of Appl2 in inflammation, the level of Appl2 was determined in the primary macrophages in response to LPS stimulation. The expression of Appl2 decreased gradually, suggesting that Appl2 may be involved. Indeed, Appl2 KO mice showed more severe endotoxin shock compared with the WT mice when challenged with LPS.

Since PI3K/Akt pathway was well known to be essential in LPS-induced acute inflammation [28] and Appl1 was shown to regulate Akt activity and substrate specificity [29], it is of interest to examine the relationship between Appl2 and Akt in inflammatory reaction. Thus, we next investigated whether the LPS-stimulated Akt pathway can be affected in the primary macrophages from the Appl2 KO mice. Interestingly, the Akt signaling was enhanced in the LPS treated primary macrophages from the Appl2 KO mice compared with that from the WT mice. These results appear to be consistent with those of previous reports, wherein Appl1 and Appl2 were able to facilitate Akt activation through yin-yang regulation in the muscle cells, and the fact that phosphorylation of Akt as well as glucose uptake were enhanced in Appl2 KO mice [24, 30]. The higher level of Akt activity may be responsible for the enhanced expression of NF-kB and subsequent more aggressive inflammatory reaction in Appl2 KO mice when challenged by LPS.

TLRs are a class of proteins that play a key role in the innate immune system as well as the digestive system. Activation of TLR signaling through recognition of pathogen-associated molecular patterns leads to the transcriptional activation of genes encoding for pro-inflammatory cytokines and chemokines by the way of PI3K/Akt, which in turn control the activation of antigen-specific adaptive immune response [31]. To understand the concise mechanism of Appl2 that regulates inflammation, we hypothesized that Appl2 may interact with the TLR4 receptor because it has been reported to increase Akt phosphorylation in a TLR4 high expression cell line [32–34]. Opposite to our expectation, binding between the Appl2 and TLR4 was not observed. MyD88 is an important protein in TLR signaling pathways by dimerizing with the TLR receptors [26].Binding between the Appl2 and MyD88 was not observed either. Some study showed that MyD88 can form a complex with PI3-kinase, an important regulatory protein of Akt [26, 35]. Therefore, Appl2 may be involved in the downstream of TLR4 pathway. As expected, PI3K apparently interacted with Appl2. These results are consistent with those of a previous paper, wherein Appl2 suppresses insulin signaling pathway and Akt phosphorylation in a PI3K-dependent manner [30]. Our results also showed that Appl2 can interact with Appl1when challenged by LPS. These results indicate that a complex formed by Appl1, Appl2 and PI3K likely exists, and Appl2 has a negative effect on the activation of PI3K/Akt pathway and therefore, the expression of downstream genes involved in inflammation.

In our experiment, Appl2 KO mice exhibited symptoms of more severe endotoxin shock and increased production of serum proinflammatory cytokines when challenged with LPS. Although the precise mechanism is not known at this point, it is perceivable that absence of Appl2 enhances the interaction between Appl1 and PI3K and hence further activates the Akt signaling pathway and subsequent inflammatory responses. Indeed, deletion of Appl1exhibited characteristics of reduced Akt activation and decreased inflammatory response.

## Conclusions

Findings of the present study show that Appl2 is an important negative regulator in PI3K/Akt-mediated NF-kB activation and therefore serves as a target with great therapeutic potential to curb inflammation.

## Methods

### Generation of Appl1 and Appl2 KO mice

Generation of Appl1 and Appl2 KO mice has been described previously [30, 36]. Briefly, a 4.7 kb genomic fragment upstream of mouse Appl1 exon 17 and a 4.2 kb genomic fragment downstream from Appl1 exon 18 were used as the recombination arms in the construction of the targeting vector. A neomycin-resistance gene (neo) expression cassette was inserted between the two regions, which resulted in a vector designed to delete the two RNA-binding motifs in an exon 17 and 18. A 3.4 kb and a 3.6 kb genomic fragment upstream and downstream of mouse Appl2 exon 5 were used as recombination arms, respectively, in the construction of the targeting vectors. A neocassette with the loxP sequence was inserted into the upstream of exon 5. Another loxP site was cloned into downstream of exon 5. Both the two targeting vectors were performed according to protocol: C57BL/6 J embryonic stem (ES) cells were electroporated with the linearized targeting construct [37, 38]. After selected by G418, clones with targeted alleles were identified through polymerase chain reaction (PCR) analysis. ES cells with targeted alleles were injected into the blastocysts of C57BL/6 J mice. The chimeric males were mated with the females of the same strain to obtain heterozygous mutant mice. The Appl1 mice intercrossed to obtain homozygous and wild-type (WT) mice. Then the Appl2-floxed mice were crossed with EIIA-Cre mice, and the homozygous Appl2 KO mice were subsequently generated from these mice.

### Animal and ethics statement

Male APPL1/2 KO mice in C57BL/6 background and WT mice were housed in standard specified pathogen-free mice room and maintained on a 12 h light–dark cycle at 22 ± 2°C and fed standard chow (15.9 kJ/g, 10% of energy as fat, 20% of energy as protein, 70% of energy as carbohydrate). Humane endpoints of animal behaviors were used to minimize suffering in the survival and animal studies. All intraperitoneal injections and sacrifice for organ removal and peritoneal macrophages isolation were performed under anesthesia by sodium pentobarbital (50 mg/kg, i.p.). All of the experimental procedures were carried out in accordance with the NIH Guidelines for the Care and Use of Laboratory Animals. Animal experiments were approved by the Animal Care and Use Committee of Guangzhou Institute of Biomedicine and Health, Chinese Academy of Sciences.

### Survival studies

Male mice (8 weeks old) weighing 21 ± 2 g were treated once with intraperitoneal injection of LPS (Sigma-Aldrich) one time. The Appl2 KO and WT mice were injected with a dose of LPS at 20 mg/kg weight (n = 9). The mice were returned to their cages after LPS injection and closely monitored for their behaviors during the experiments every eight hours. Mice were given *ad libitum* access to food and water at all times. In case clinical signs of distress or moribund were recognized, animals were euthanized by sodium pentobarbital (50 mg/kg, i.p.) followed by cervical dislocation. Clinical signs of endotoxin shock include reduced locomotion, signs of diarrhea, piloerection and body weight loss. The number of survival mice was recorded at the time points of 24 h, 48 h, 72 h after LPS challenge, and the percent survival rates were calculated from the number of animals that survived by the total number of tested animals at the given time. The Appl1 KO and WT mice were injected with a dose of LPS at 30 mg/kg weight (n = 9), and the survival rate experiment was performed as above.

### Serum and tissue samples

Appl1/2 KO and WT control mice were injected i.p. with LPS as described. Whole blood samples were collected into tubes containing an anticoagulant by tail incision at the time points of 1 h, 12 h, 24 h, 48 h, 72 h after LPS challenge and incubated at room temperature for 15 min prior to centrifugation at 3,000 × g for 20 min. The tail wounds of mice were wiped by the 70% alcohol cotton ball to prevent infection. The serum was extracted and stored at −80°C before processing for cytokine assays.

### Histochemistry

The mice were euthanized by cervical dislocation under anesthesia by sodium pentobarbital (50 mg/kg, i.p.) after 24 h by LPS injection, and the kidney tissues were fixed in 4% formaldehyde overnight at room temperature immediately after the mice were sacrificed. Tissues were paraffinized and sectioned by microtome, and the slides were stained with hematoxilin and eosin (HE) (Sigma) following the standard protocol. Sections were examined by light microscopy.

### Isolation and culture of peritoneal macrophages for in vitro studies

Peritoneal macrophages from C57BL/6 mice and Appl1/2 KO mice were collected as previously described [39]. Briefly, peritoneal macrophages were elicited by an intra-peritoneal injection of 2 ml of 4% thioglycolate (Gibco) in distilled water. After 4 days, the elicited macrophages were collected by peritoneal lavage with 10 mlof Hank's balanced salt solution (HBSS). The peritoneal lavage fluids were centrifuged at 1500 rpm for 5 min and the cells were re-suspended in RPMI-1640 medium (Invitrogen) supplemented with 10% heat-inactivated fetal calf serum, 100 units/ml penicillin, and 100 μg/ml streptomycin. Equal numbers of peritoneal macrophages derived from three mice were pooled and seeded in three wells (5 × 10^5^ cells/well). Peritoneal cells were incubated at 37°C in an atmosphere of 5% CO_2_ for 3 h to allow the peritoneal macrophages to adhere. Non-adherent cells were removed by washing with PBS twice and the attached cells were used as peritoneal macrophages.

### Quantification of cytokine expression

Serum cytokine levels and cytokines secreted from primary macrophages were examined through ELISA (R&D). The macrophages were stimulated with 1 μg/mL LPS (Sigma) at specified times and the supernatant was collected for quantification of cytokine (TNFα and IL-1β) expression.

### Western blot analysis

Peritoneal macrophages were treated with 1 μg/mL LPS for 20 minutes. Peritoneal macrophages were washed with ice-cold PBS, and proteins were extracted from the cells in a RIPA buffer (Beyotime). The concentration of protein in the lysate was determined using a Bradford assay (Bio-Rad). The cell lysates were resolved through SDS-PAGE and transferred onto a polyvinylidene fluoride membrane. The membrane was blotted with antibodies to Akt (Cell Signaling), p65 (Cell Signaling), IκB (Cell Signaling), IKKβ (Cell Signaling), phospho-Akt (Cell Signaling), phospho-IKKβ (Abcam), phospho- IκBα (Cell Signaling), phospho-p65 (Cell Signaling), Appl1, and Appl2. Appl1 and Appl2 antibodies were from Aimin Xu.

### Immunoprecipitation

Peritoneal macrophages were treated with 1 μg/mL LPS for 20 min. The cells were washed with PBS and proteins were extracted via treatment with RIPA buffer (Beyotime) at 4°C for 30 minutes. Cells were centrifuged at 10,000 g for 30 minutes at 4°C, allowing cell debris to be pelleted and discarded. Cellular protein (100 μg) was mixed with 1 μg of anti-Appl1 or Appl2 antibody and incubated over-night at 4°C. Then, 10 μl of protein G Plus-agarose (Santa Cruz) was added to these samples and incubated for another 4 h at 4°C. After the incubation, samples were washed three times with lysis buffer. The washed samples were re-suspended in SDS sample buffer (Beyotime) and heated at 100°C for 5 min prior to electrophoresis.

### Statistical analysis

In order to examine the survival rate of APPl1/2 KO and WT mice when challenged by LPS, a Kaplan-Meier analysis was performed using terminal mortality as the endpoint. Data were presented as mean ± SD for the statistical comparison of the two samples. Student’s *t*-test was used for evaluation. Survival curves were generated using the Kaplan-Meier method, and their significance was evaluated using the Log-rank test.

## Abbreviations

IL-1β: Interleukin 1β
NF-κB: Nuclear factor κB
TNF-α: Tumour necrosis factor-α
WT: Wild-type
Appl1/2: Adaptor protein containing the pleckstrin homology domain, phosphotyrosine domain, and leucine zipper motif 1/2
Akt: Protein kinase B
IKK: IκB kinase
TLR4: Toll-like receptor4
MyD88: Myeloid differentiation factor88
LPS: Lipopolysaccharide
P65: Nuclear factor NF-kappa-B p65 subunit
PI3K: Phosphatidylinositol 3-kinase
P85α: The p85 subunit of PI3K.
